# The role of iron in neurodegenerative disorders: insights and opportunities with synchrotron light

**DOI:** 10.3389/fphar.2014.00191

**Published:** 2014-08-19

**Authors:** Joanna F. Collingwood, Mark R. Davidson

**Affiliations:** ^1^Warwick Engineering in Biomedicine, School of Engineering, University of WarwickCoventry, UK; ^2^Materials Science and Engineering, University of FloridaGainesville, FL, USA; ^3^The Tech Toybox, GainesvilleFL, USA

**Keywords:** iron, synchrotron X-rays, human brain, Alzheimer’s disease, Parkinson’s disease, neuromelanin, amyloid aggregation, magnetic resonance imaging

## Abstract

There is evidence for iron dysregulation in many forms of disease, including a broad spectrum of neurodegenerative disorders. In order to advance our understanding of the pathophysiological role of iron, it is helpful to be able to determine in detail the distribution of iron as it relates to metabolites, proteins, cells, and tissues, the chemical state and local environment of iron, and its relationship with other metal elements. Synchrotron light sources, providing primarily X-ray beams accompanied by access to longer wavelengths such as infra-red, are an outstanding tool for multi-modal non-destructive analysis of iron in these systems. The micro- and nano-focused X-ray beams that are generated at synchrotron facilities enable measurement of iron and other transition metal elements to be performed with outstanding analytic sensitivity and specificity. Recent developments have increased the scope for methods such as X-ray fluorescence mapping to be used quantitatively rather than semi-quantitatively. Burgeoning interest, coupled with technical advances and beamline development at synchrotron facilities, has led to substantial improvements in resources and methodologies in the field over the past decade. In this paper we will consider how the field has evolved with regard to the study of iron in proteins, cells, and brain tissue, and identify challenges in sample preparation and analysis. Selected examples will be used to illustrate the contribution, and future potential, of synchrotron X-ray analysis for the characterization of iron in model systems exhibiting iron dysregulation, and for human cases of neurodegenerative disorders including Alzheimer’s disease, Parkinson’s disease, Friedreich’s ataxia, and amyotrophic lateral sclerosis.

## INTRODUCTION

The study of iron in neurodegenerative disorders emerged a century ago, arguably catalyzed by developments in histological methods to demonstrate iron in tissue, and by observations of abundant iron deposition in the human brain in health and disease. Lhermitte et al. (1924) describe a Parkinson’s disease (PD) case with diminished intracellular iron and iron-rich deposits (“globules”) in the globus pallidus (GP), and with iron in the substantia nigra (SN) apparently unaltered. Yet on the 90th anniversary of this paper, as we see evidence that iron chelation can modify brain iron with beneficial effects for PD patients (Devos et al., 2014), there remain open questions and continuing debate about the precise nature and consequence of iron dysregulation in PD (Gerlach et al., 1994; Galazka-Friedman et al., 1996; Oakley et al., 2007; Friedman et al., 2009; Crichton et al., 2011; Visanji et al., 2013). Similar questions about the pathophysiology of iron arise in many neurodegenerative disorders including multiple system atrophy (MSA; Visanji et al., 2013), Alzheimer’s disease (AD; Smith et al., 2010), Huntington’s disease, Friedreich’s Ataxia, and motor neuron disease (MND) including amyotrophic lateral sclerosis (ALS). The extensive evidence for poorly liganded iron contributing to the pathophysiology of neurodegenerative disorders is reviewed elsewhere (Kell, 2010).

Disruptions to normal iron homeostasis can exacerbate deleterious excess formation of radical species (Kell, 2010), and changes in regional or cellular iron concentration, or in the proteins responsible for tightly regulating iron metabolism, may indicate vulnerability to oxidative stress damage that is observed in the pathophysiology of neurodegenerative disorders (Smith and Perry, 1995; Castellani et al., 2007; Rouault, 2013). Aberrant peptide aggregation leads to the formation of pathological hallmarks of neurodegenerative disorders such as the beta amyloid (Aβ)-rich plaques in AD, and the alpha (α-)synuclein-rich Lewy bodies in PD. Iron has long been implicated in mechanisms of toxicity associated with aberrant peptide aggregation (Rottkamp et al., 2001), although work to determine a precise role for iron is ongoing (House et al., 2004; Everett et al., 2014a). The reductase behavior of the Aβ and α-synuclein peptides has been shown *in vitro* (Khan et al., 2006; Davies et al., 2011). The chemical reduction of ferrihydrite (iron oxide) particles by Aβ_42_ has also now been demonstrated *in vitro* (Everett et al., 2014a). The contribution of synchrotron X-ray analysis to such studies, and to understanding fundamental structural and conformational properties of the peptides, will be considered here.

The use of X-rays to study iron in human tissue, and proteins responsible for iron homeostasis, is well-established. For example, X-ray fluorescence (XRF) spectroscopy was used in 1968 to evaluate iron content in formalin-fixed tissues (Earle, 1968). The use of X-rays for metallomics-related research has been advanced by the design and development of synchrotron facilities: particle accelerators with a cyclical path that generate intense beams of light primarily in the X-ray region of the electromagnetic spectrum. The first synchrotrons were designed in the 1940s, and subsequently built as large-scale facilities that over several generations of development have enabled exceptionally diverse and cutting-edge research. In the past two decades there has been significant progress in improving beam focusing and detectors, facilitating chemical element imaging and analysis at micro- and nanometer spatial resolution using techniques such as synchrotron XRF (SXRF) and X-ray absorption near edge spectroscopy (XANES). There has also been progress in developing techniques to analyze proteins and protein–metal interactions including high-throughput crystal structure analysis of proteins, advancing methods to observe conformational changes to proteins as a function of temperature and pH, for example, and techniques permitting time-resolved analysis of these processes (Duke and Johnson, 2010).

Perspectives on developments over the past decade are given in several reviews of methods for the spatial analysis of metals in biological tissues: these consider both stand-alone laboratory instruments and large shared facilities including neutron spallation and synchrotron light sources. Lobinski et al. (2006) reviewed various methods for the analysis of trace metals in biological environments, noting how technical advances such as nanoflow chromatography enabled work with significantly smaller sample volumes, and how improvements in the delivery of focused synchrotron beams significantly increased scope to analyze the local chemical environment of metal ions with X-ray absorption spectroscopy (XAS). Synchrotron methods including Fourier transform infrared (FTIR) micro-spectroscopy and imaging (Miller and Dumas, 2006), scanning transmission X-ray microscopy (STXM; Obst and Schmid, 2014), and SXRF microscopy (Ralle and Lutsenko, 2009) have been considered, while Jackson reviewed the stand-alone technique of laser ablation inductively coupled plasma mass spectrometry (LA-ICP-MS) for transition metal element analysis in rodent brain (Jackson et al., 2006), and Qin et al. (2011) compared SXRF microscopy with LA-ICP-MS and secondary ion mass spectrometry (SIMS). Notably, McRae et al. (2009) produced an exceptionally broad and thorough review of techniques for *in situ* imaging of metals in cells and tissues.

Synchrotron access is necessarily limited, and experiment lead-time from proposal to beamtime is typically six to nine months. The techniques provide opportunities to advance presently intractable questions, and many of the analytical techniques available are highly complementary, require minimal sample preparation, and may be performed with little or no significant damage to the sample. For technical resources and news of developments at individual synchrotrons, readers are referred to the most recent articles cited in this review, and to the website www.lightsources.org/. Here, we provide some perspective on developments in the field over the past twenty years, drawing on examples to show how pioneering experiments in synchrotron X-ray research have contributed to our understanding of the role of iron neurobiology, and by extension its role in various neurodegenerative diseases.

## DETERMINING IRON DISTRIBUTION AND FORM IN TISSUES AND CELLS

Iron is the most abundant of the transition metals in human brain, but to gain a full understanding of how iron in cells and tissues is compartmentalized and bound requires exceptional analytical sensitivity and specificity. While chemical and immunohistochemical methods have been successfully used to progress understanding of iron and other metal elements at regional, cellular, and sub-cellular level, they have limitations which have prompted investigators to develop alternative techniques with greater sensitivity, specificity, and spatial resolution for the analysis of metal ions in tissues. The majority of systems are laboratory-based, including particle-induced X-ray emission (PIXE), electron probe X-ray microanalysis (EPMA), and LA-ICP-MS, reviewed in depth elsewhere (McRae et al., 2009). Within the bio-iron community, arguably the best-known synchrotron X-ray technique for iron analysis in tissues is SXRF, which has been demonstrated at increasingly high spatial resolution and rapid analytical rates to map cells and tissues in recent years (Collingwood et al., 2005a; Tomik et al., 2006; Ortega et al., 2007; Popescu et al., 2009a; Leskovjan et al., 2011; Ugarte et al., 2012).

### SXRF MAPPING OF IRON IN TISSUES AND CELLS

**FIGURE 1:**
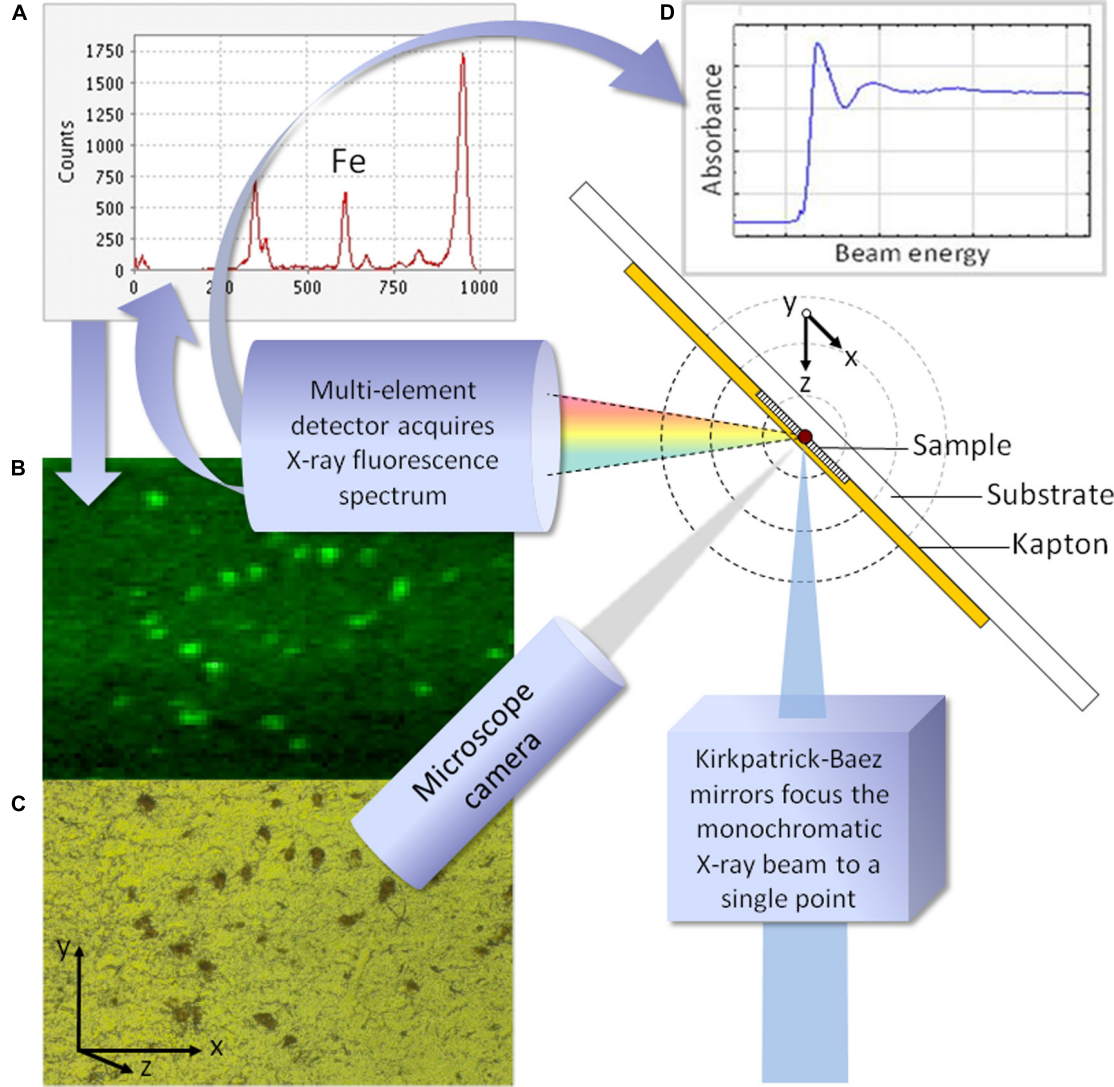
**Schematic illustrating a typical configuration for synchrotron X-ray fluorescence analysis.** For fluorescence mapping (synchrotron XRF, SXRF) the incoming beam is fixed in energy, and the sample is moved incrementally through the plane described by x and y. In panel** (A)**, the full fluorescence spectrum is obtained at each point, and used to generate element specific maps. Panel **(B)** shows the iron map for the region of pigmented substantia nigra shown, corresponding to the region viewed via the microscope camera **(C)**. To obtain the X-ray absorption spectrum from a point of interest in the sample, shown in panel **(D)**, the beam energy is varied and the XANES spectrum recorded.

When an X-ray beam interacts with a sample, it excites natural fluorescence from the chemical elements within the tissue for which the fluorescence excitation energy is at or below the incident beam energy. The fluorescent X-rays have element-specific wavelengths, and their intensity determines the relative abundance of each element. XRF was initially used with laboratory systems to capture spectra from homogenized samples, but the beam intensity and fine optics control delivered by synchrotron facilities has enabled SXRF to evolve into a technique for high-resolution mapping of samples. The peak intensity for specific elements, or the full fluorescence spectrum, may be obtained in each map pixel (**Figure 1**). SXRF emission is directly proportional to atomic abundance, so in principle SXRF can be used for quantitative analysis. SXRF mapping in biological samples has mainly been semi-quantitative to date, where challenges with matrix-matching, and localized foci of specific elements causing heterogeneous patterns of self-absorption, has made true quantification impractical. The penetration depth of the beam is typically ≤1 mm, so that for the majority of cell or tissue samples the beam will penetrate the full thickness and beyond; an exception to this being the use of thick samples in rapid SXRF (Popescu et al., 2009a). In the focused beam configuration it has become standard to map with resolution better than 100 nm at some beamlines; for XANES it continues to be typical to work at micron-scale spatial resolution.

### X-RAY ABSORPTION SPECTROSCOPY ANALYSIS WITH XANES AND EXAFS

Determining the oxidation state and structural environment of iron in biological materials is a significant analytical challenge; more so if sample microstructure is to be preserved. Synchrotron XAS is an attractive candidate to address questions about the chemical state and mineralized form of iron in cells and tissues, because of the exceptional sensitivity and spatially resolved precision with which microfocus XAS can be performed. XANES provides chemical state information, and can be used to identify the primary forms of iron oxide and/or iron-binding metalloproteins in the sample volume from which the spectrum is obtained (**Figure 1**). For high-quality spectra (from, for example, purified samples of iron-binding proteins), the energy spectrum may be collected over an extended range, to obtain extended X-ray absorption fine structure (EXAFS), and the data utilized to extract fundamental structural information about the local environment. EXAFS can reveal the identities and structural positions of atoms surrounding the scattering iron atoms: for example, the neighboring shells of oxygen and iron atoms to determine the types of iron oxide found in ferritin, hemosiderin, and neuromelanin.

The regions of the fluorescence energy spectrum used in SXRF mapping, XANES, and EXAFS are illustrated graphically in a review of the analysis of metalloproteins in cells (Cook et al., 2008), and below we consider the application of SXRF imaging and XANES to analyze iron in various neurodegenerative disorders.

## X-RAY FLUORESCENCE ANALYSIS OF IRON IN NEURODEGENERATIVE DISORDERS

X-ray fluorescence was used to analyze iron in neurodegenerative disorders as early as 1968, using a laboratory X-ray source (Earle, 1968). XRF spectra showing elemental intensity as a function of angle, determined by fluorescence emission energy, were obtained from the brain tissues of 11 PD cases and an unknown number of controls with no known neurological conditions. Emission peak intensities were compared to obtain preliminary indications about the concentration of iron and other elements in regions of the brain. Detection limits were judged to range, for light to heavy elements, respectively, from approximately 100 to 10 μg element/g dried tissue. This enabled specific and simultaneous analysis of physiologically important elements including the transition metals iron, zinc, and copper. Dissection and pelleting of samples prior to analysis meant that information about iron distribution in the tissue microstructure was not preserved. However, the heterogeneous and reproducible patterns of brain iron deposition enabled comparisons of regional iron levels for health and disease. Earle concluded that iron was consistently elevated in PD brain tissue compared for control. As Earle found elevated brain iron in a case of Pick’s disease, as well as in an elderly man with a recent infarct, the brain iron changes were not attributed as being specific to PD. Earle acknowledged the limitations of using archived formalin-fixed tissues (see Section “Approaches to Study Design and Sample Preparation”), some of which may have been in storage since 1862, and proposed that the experiments be verified with unfixed tissue. This was done by others in due course, including Dexter et al. (1989) and Jenner (1989).

### SXRF: THE EMERGENCE OF SYNCHROTRON X-RAY FLUORESCENCE MAPPING

Synchrotron XRF provides orders-of-magnitude higher flux than can be achieved with bench-top X-ray systems. This, combined with developments in optics to focus high intensity X-rays into micrometer or nanometer diameter beams, significantly improved the signal that could be obtained from small sample volumes. It became practical to obtain SXRF spectra from multiple points in a sample, and to automate this process to obtain maps of cells and tissues, with each pixel containing a complete metal-ion spectrum (**Figure 1**). Synchrotron beamlines utilizing soft X-rays now routinely achieve sub-micron, and in some cases ∼10 nm spatial resolution. This energy range is better-suited to light elements, and for combined iron-fluorescence and absorption analyses it is usually more efficient to work at beamlines with access to the K-edge for iron.

Ektessabi et al. (1999) collected SXRF spectra from dopaminergic neurons in single cases of healthy control and PD brain, to investigate elemental constituents, concentrations, distributions, and chemical states of iron and other elements in the individual neurons. Mapping resolution was 6 μm × 8 μm, and incident beam energy 13.5 keV. Areas ∼ 100 μm × 100 μm were mapped in formalin-fixed tissue sectioned at 8 μm thickness prior to mounting on Mylar film. Consistent with prior XRF observations in pelleted samples (Earle, 1968), they observed iron signal associated with the neuromelanin granules to be approximately twice as intense in PD tissue compared to the control. Neuromelanin-associated iron co-localized with other metals including calcium, zinc, and copper. Efforts were made to determine the iron oxidation state, but limited flux in the microfocus configuration prevented useful XANES being obtained from such dilute samples (Ektessabi et al., 1999). Comparisons of iron signal within and without the neuromelanin-rich neurons led to a reported ratio of 11:1; this result in formalin-fixed tissue is very different to results from a subsequent independent study where we observed a ratio ∼ 3:2 for neuron versus neuropil analysis in unfixed specimens measured by EPMA, which then became ∼1:1 when iron counts were normalized to sulfur counts (Oakley et al., 2007). One interpretation of this difference is that extra-cellular (or weakly bound) iron may have been selectively leached from the formalin-fixed tissue; however, the evidence in Ektessabi’s paper for raised intra-neuronal iron in PD compared to healthy control is in good agreement with our work in the unfixed tissues which confirmed raised iron for PD versus control (significant at *p* < 0.0001) in individual dopaminergic neurons using chemically unfixed specimens from 16 PD cases and 14 controls without neurologic disease (Oakley et al., 2007).

Subsequent to Ektessabi’s study, Szczerbowska-Boruchowska et al. (2004) performed some of the earliest SXRF analysis on unfixed tissues from neurodegenerative disorders, comparing iron distributions in SN and spinal cord tissue from single cases of PD and ALS and a control with no neurological disorder. They worked with fresh-frozen 20 μm cryosections that were freeze-dried following mounting onto a plastic (AP-1) foil. Adjacent tissue sections were used for supporting histology. Areas 500 μm × 500 μm were mapped at 10 μm × 5 μm resolution, with higher resolution mapping (5 μm × 2 μm) of pigmented neuronal cell bodies in the SN. With a dwell time of 3 s per point, detailed maps could be obtained in a matter of hours. Relative concentrations may be well-determined by SXRF, where sample preparation and measurement conditions (such as detector position) are identical, and data are normalized to incoming beam. In the present study, standards were used to obtain apparent elemental concentrations, and detection limits were determined using the following expression developed in keeping with the original framework set out by Currie (Currie, 1968):

$$D\,L_{i}=3.3\,\frac{C_{i}}{Y_{l}}\,\sqrt{B_{i}}$$

where the detection limit DL_i_ depends on the mass per unit area *C_i_* for element *i*, the net peak area of element *Y_i_* for element *i*, and the background *B_i_*.

Szczerbowska-Boruchowska et al. (2004) observed that iron, along with Zn, S, and Cl, was concentrated in the SN neuronal cell bodies regardless of disease state. They observed, consistent with the prior study in fixed tissue (Ektessabi et al., 1999), elevation of Fe (and other elements) in the PD SN compared to the healthy control, seeing iron elevation in both nigral neuronal cell bodies (perikarya) and white matter. It was noted that iron was “*strongly accumulated inside neuron perikaryal parts and, additionally in the case of PD, in structures that were not identified histopathologically*” (Szczerbowska-Boruchowska et al., 2004). The latter may have included extracelluar deposits (perhaps of neuromelanin) or iron-rich glia. Neurons in the spinal cord, as compared to the SN, did not carry the same typical elevation of Fe, but – in addition to other elements including Ca and Zn – the apparent Fe concentration increased in ALS spinal cord white matter compared to control.

While this study only included single cases, it is reportedly the first micron-level-precision spatial analysis of Fe focii in SN neurons to be performed in chemically unfixed tissue; an important step after the confirmation by ICP-MS (in chemically unfixed bulk tissue samples) of elevated iron in PD SN compared to healthy controls (Dexter et al., 1989). Subsequent examination of spinal cord using a slightly expanded number of ALS (*n* = 3) and control (*n* = 5) cases found variable levels of Fe in the spinal cord neuron perikarya and in the white matter. No significant differences were observed at whole tissue level (Tomik et al., 2006). The heterogenity of metal ion distribution in many atomical structures, and the associated length scales, means that SXRF mapping is not always an ideal tool for whole tissue comparisons unless anatomical regions are very well matched: this is equally a challenge for large-area rapid SXRF (Popescu et al., 2009a) as it is for microfocus studies. Here, prior observations had been made of elevated iron and selenium in lumbar spinal cord from 38 MND cases compared with 22 controls using bulk samples for neutron activation analysis (NAA). The marked elevation of Fe and Se, coupled with the absence of correlation with disease stage or motor neuron counts, prompted the conclusion that the Fe and Se elevation is an early stage event in the disease process and likely a contributing factor (Ince et al., 1994; Markesbery et al., 1995).

**FIGURE 2:**
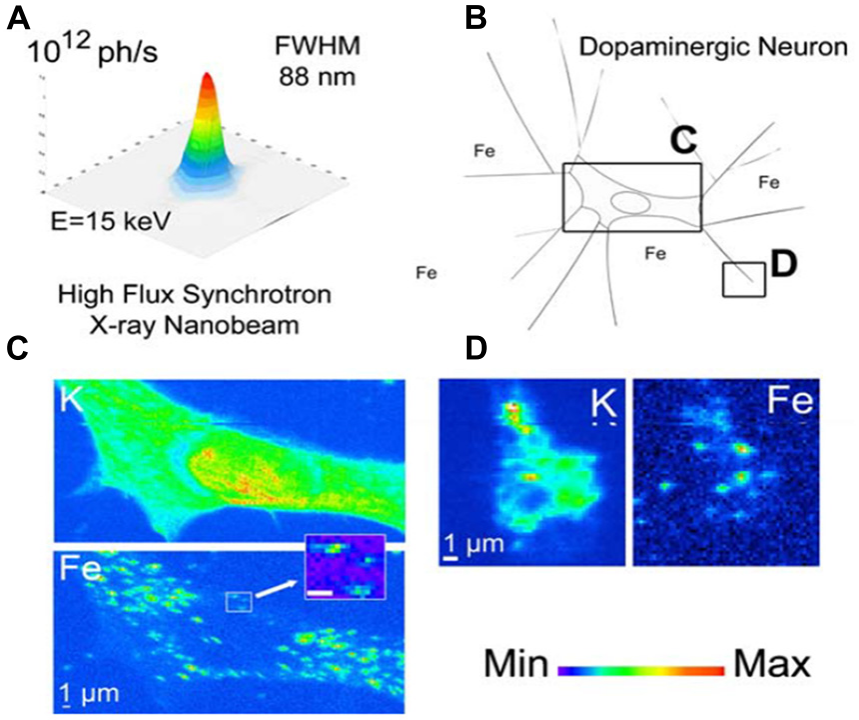
**SXRF mapping with hard X-rays at sub-cellular resolution.** Dopamine-producing cells are chemically mapped with sub-90 nm beam, revealing potassium and iron distribution. In panel **(A)** the intensity distribution in the focal plane is shown; panel** (B)** illustrates the dopamine producing cells exposed *in vitro* to 300 mM FeSO_4_ for 24 h. Chemical element distributions of potassium (K) and iron (Fe) were recorded on distinct cellular areas including cell bodies **(C)** and distal ends **(D)**. Iron was found in 200 nm structures in the cytosol, neurite outgrowths, and distal ends, but not in the nucleus. Units for the min-max intensity bar are arbitrary. Scale bars = 1 mm. Adapted from Ortega et al. (2007).

A strength of SXRF mapping is its ability to sensitively detemine relative spatial distributions of elements at a wide range of resolutions ranging from small anatomical regions and cell layers to the sub-cellular. Ortega et al. (2007) showcased sub-micron SXRF by achieving 90 nm resolution mapping with an extended version of the Kirkpatrick-Baez (KB) focussing geometry utilizing the hard X-ray energy range, as shown in **Figure 2**. This study of PC12 rat cells was designed to extend studies of iron in the SN by looking at the relationship between iron and dopamine. The PC12 pheocromocytoma cell line served as an *in vitro* model of dopaminergic neurons; cells were differentiated with nerve growth factor prior to exposure to sub-cytotoxic concentrations of iron and/or AMT (alpha-Methyltyrosine), an inhibitor of dopamine synthesis. The cells were freeze-dried prior to measurement, and SXRF sampling times were 1/3–1 s per point with repeat sampling, rather than extended dwell times, to achieve good signal:noise. This is consistent with recommendations for optimal preservation of metal ion information in microfocus SXRF analysis of biological materials (Bacquart et al., 2007). By utilizing a combination of epi-fluorescence to visualize dopamine, and SXRF mapping for iron, they observed the co-localization of iron and dopamine in dopamine vesicles, and demonstrated the impact of dopamine synthesis inhibition on iron distribution. Use of sub-micron resolution SXRF allowed detailed visualization of neuronal processes in addition to the perikarya, revealing that blocking dopamine synthesis led to lowering of iron content specifically in dopamine vesicles, predominantly in the neuronal processes.

### THE ADVENT OF QUANTITATIVE SXRF

The above study illustrated a significant advance in synchrotron hard X-ray capabilities, with a factor of 10 improvement in SXRF imaging resolution, and a reported detection limit of 10^-18^ g of Fe within a 100 nm diameter structure (Ortega et al., 2007). Although SXRF is unique amongst the microprobes for its combined sensitivity and non-destructive potential, it has historically been recognized as a semi-quantitative method that demonstrates consistent but relative (not absolute) metal ion distribution when compared with quantitative microprobe techniques (Ugarte et al., 2012; O’Reilly et al., 2014). The practical challenges of SXRF analysis, including methods to analyse images and draw meaningful comparisons between samples, are explored carefully in a elemental analysis of breast tissue (Geraki et al., 2002); the study also considers challenges in SXRF calibration. In principle, quantitative analysis requires matrix-matched standards that have comparable bulk composition, density, and thickness of the sample. Due to recent efforts, SXRF mapping is now being recognized for its quantitative potential in the analysis of biological materials (Qin et al., 2011). Approximate concentrations, sufficient to permit a degree of sample comparison, have previously been obtained by use of standards or supporting analysis of the sample materials. In Ortega’s analysis of the PC12 cells, hundreds of additional cells were analyzed by microPIXE, to perform an approximate calibration for the SXRF nanoimaging via mass normalization of the X-ray emission (Ortega et al., 2007). Although the concentrations achieved are not absolute, this is a good example of how complementary analytical approaches may be used to overcome the limitations of a given technique. The PC12 cell model was subsequently analyzed using the same SXRF and microPIXE approach to investigate competition between iron and manganese (Carmona et al., 2010). Manganese toxicity can result in a parkinsonism-like disorder in humans and it has been postulated that Fe and Mn compete for serum iron binding protein transferrin, and subsequent iron transport protein divalent metal transporter (DMT), but there is conflicting evidence as to whether iron uptake is promoted or diminished by Mn exposure in PC12 cells (Carmona et al., 2010). In addition to demonstrating how Mn localizes to the Golgi apparatus, a decrease in intracellular Fe was observed after the cells were exposed to Mn, consistent with the possibility that Mn competes with Fe for binding sites and transport mechanisms (Carmona et al., 2010). An alternative approach to calibrate SXRF for elemental mapping in cells incorporates the morphological data that can be obtained from atomic force microscopy (AFM) and STXM, to correct for the self-absorption effects that can strongly distort analysis of the lighter elements such as magnesium (Malucelli et al., 2013). Meanwhile, Kosior et al. (2012) have proposed what is reportedly the first method to achieve absolute quantitative analysis of iron and other metal elements by SXRF, using quantitative phase contrast imaging to obtain the projected mass for each sample, and thereby perform the mass correction pixel-by-pixel for the SXRF maps. This is designed to address the challenges for quantification posed by factors such as the variations at the cellular level in density and cell thickness. Most SXRF analysis presently provides relative, rather than absolute, concentration maps of elements, as the additional information required to convert SXRF maps to perform the mass correction (Kosior et al., 2012) is not yet routinely acquired.

### SXRF MAPPING: DEVELOPMENTS IN THE CONTEXT OF ALZHEIMER’S DISEASE

In synchrotron studies to evaluate iron in neurodegenerative disorders, the primary target regions have been those previously demonstrated to accumulate iron, to be especially prone to degeneration, and/or to exhibit iron associated with pathological hallmarks of a given disease. Some of the earliest SXRF mapping experiments were therefore of the cortex and hippocampus, which undergo significant atrophy in AD. For example Ishihara et al. (2002) used small (3 mm × 4 mm) blocks of frozen tissue from the superior temporal gyrus of two confirmed AD cases, briefly fixed in 4% paraformaldehyde and 2% glutaraldehyde for 2 h prior to cryosectioning at 5 μm thickness and mounting on polyester (Mylar^®^) film. Retrospectively, hematoxylin, and eosin staining was used to determine tissue structure (Ishihara et al., 2002). The small areas and absolute numbers of cells sampled, in combination with the absence of a control for the AD cases, limit the conclusions that can be drawn from this study. The fixation step also raises the possibility that loosely bound metal ions may have been mobilized and displaced. However, this cellular-resolution multi-elemental mapping of neuronal perikarya in the cortex is an early demonstration of how SXRF was introduced to study transition metal ion distributions in brain regions exhibiting significant pathology. SXRF was used during this same period to compare elements including iron, zinc, and copper in frozen unfixed rat brain tissue, as part of a study on iodine deficiency (Zhang et al., 2002). Relative concentrations for hippocampus versus cerebral cortex were plotted, but the structures within cortex and hippocampus were not spatially resolved. As observed for iron using histochemical methods (Morris et al., 1994), and more recently for a range of metals using microbeam analysis, the layers within the hippocampus exhibit distinct patterns of iron and zinc distribution. The zinc elevation in the dentate gyrus has been illustrated by SXRF in rat hippocampus (Flinn et al., 2005), and the iron-rich layers in the layers of the surrounding cornu Ammonis have been shown by SXRF in human hippocampus (Antharam et al., 2012). It is of interest to determine these spatial distributions of transition metals in the hippocampus, which is especially prone to atrophy in AD, as analytical studies of bulk tissue iron content in unfixed human hippocampus have produced conflicting results as to whether iron concentration in the AD hippocampus is elevated compared with healthy controls (Schrag et al., 2011).

### RAPID SCANNING SXRF

As the spatial resolution limits for hard X-ray SXRF of single cells have been pushed in recent years (Ortega et al., 2007), so have the rates at which large sample areas can be imaged (Popescu et al., 2009a,c). The time required to map a sample is primarily dependent on matrix size and the dwell time (or effective dwell time, for raster scanning) per point in the matrix. It would usually be desirable to map at a minimal resolution ∼50 μm, preferably higher, to permit delineation of primary cell layers if not individual cells. For a tissue section cut with thickness ∼20 μm, and with typical elemental concentrations requiring ∼1 s per point (including overhead) to obtain useful fluorescence signal, it would therefore take a little over 10 h to map a 10 mm × 10 mm area. Use of synchrotron experiment time (beamtime) is often optimized by minimizing the area required for scanning, and working at the lowest resolution that permits the experiment aims to be achieved. However, Nichol and colleagues have looked to overcome the limited sample area that can be analyzed by SXRF by developing an approach using very thick (conventional autopsy) samples. This maximizes the opportunity for the incoming beam to interact with the sample and thereby maximizes fluorescence yield from each anatomical region of interest, subject to any self-absorption effects. As Popescu et al. (2009a) observe, the escape depth is element-dependent, so although under most conditions they can obtain relative metal ion distribution maps across a sample area, the penetration depth for each map will differ as a function of the element mapped. Their approach, utilizing thick tissue slices and rapid SXRF scanning, has enabled transition metal maps to be generated for selected regions of the central nervous system in cases of PD, spinocerebellar ataxia, and healthy control (Popescu et al., 2009a,b,c). This is an advance on histochemical staining in that the elemental maps are achieved simultaneously from a given sample, and there is excellent specificity (if spectra are fitted, rather than simply gated), for elements in addition to iron such as copper and zinc, so long as the element-specific differences in sampling depth are not critical to the experiment design. Of the transition metals in brain tissue, Fe is the most abundant on average, followed by Zn, and then Cu. One of the sacrifices of rapid SXRF (which also arises in more conventional microfocus SXRF measurements) is that in order to minimize scan time, the signal:noise for the individual pixel spectra are usually insufficient to gather robust data about other more dilute metal ions present in the tissue such as Mn, as typically occurs when working with an effective time of <6 ms per 40 μm pixel. This can be addressed by reducing the scan rate to increase the effective dwell time per pixel (although this must be limited above a certain value to avoid beam damage), or by repeating scans (Bacquart et al., 2007). Although high effective in-plane resolution can be achieved with rapid SXRF, the beam penetrates comparatively deeply into the sample at each point in the matrix, so that a single pixel represents a small surface area but a significant depth [1/e attenuation of the Fe signal is quoted as 310 μm for brain tissue (Hopp et al., 2010)]. The angle between the incoming beam path and the fluorescence detector is 90^∘^, and each is, respectively, at 45^∘^ to the sample (see general illustration in **Figure 1**). This path must be considered if trying to define boundaries in order to undertake any high resolution multi-model analysis of SXRF maps from thick samples. Each SXRF approach presents constraints for sample preparation; for the rapid SXRF which uses large-area thick sections, samples have been fixed through immersion in formalin, which others have reported provides a vector for redistribution or loss of loosely bound metal ions (e.g., Szczerbowska-Boruchowska et al., 2004; Schrag et al., 2011). Variations in effective sampling depth occur as a function of element, but also if there are significant variations in the matrix (for example, due to calcifications, intense foci of the element being mapped, or variable hydration states). Fixed tissues have been protected from dehydration by spraying the 1 mm-thick slices with buffered formalin and heat-sealing under thin Mylar^®^ prior to mapping (Hopp et al., 2010).

The challenge of sample matching, in order to make comparisons between cases, is present for both rapid and microfocus SXRF studies, albeit at different length scales. Where practical, advance sectioning to ensure equivalent anatomical levels can significantly improve the comparisons that can be made. A rapid SXRF study comparing a case of PD (male, 70 years old) and healthy control brain (female, 80 years old) reveals many shared structures, although the levels in the coronal sections are slightly offset as described in the study and evidenced in hippocampus profiles (Popescu et al., 2009a). The PD midbrain exhibits a higher concentration of iron in the SN than for the control (Popescu et al., 2009a), consistent with prior observations. The midbrain section is taken at the level of the inferior colliculi so the iron-rich red nucleus is not observed. The extent to which offsets in level permit or preclude comparison will depend both on the degree of heterogeneity of iron deposition in a given brain region, and on the magnitude of differences between the study groups.

### VALIDATING MRI MEASUREMENT OF BRAIN IRON

As interest grows in the capacity of MRI to clinically analyse brain iron (Drayer et al., 1986; Schenck and Zimmerman, 2004), there is increasing need for direct validation of the indirect evidence for iron contrast in MRI magnitude and phase data. Correlation of MRI transverse relaxation with tissue iron concentration from bulk tissue samples has previously been achieved for AD and control brains (House et al., 2008), but a number of factors, including myelin, compete with iron to influence fundamental MRI parameters. As there is significant heterogeneity in the microstructural distribution and bound states of iron [as observed in hippocampus (Antharam et al., 2012) and SN (Blazejewska et al., 2013)], there is value in being able to directly describe the spatial relationship between iron and MRI. Given the limited sensitivity of routine iron histochemistry for this purpose [recently noted in postmortem analysis of iron and MRI in the SN (Blazejewska et al., 2013)], there have been growing efforts to correlate SXRF microscopy maps with the corresponding MRI data from post-mortem human tissue (Collingwood et al., 2008a; Hopp et al., 2010; Antharam et al., 2012). This has included studies in formalin-fixed tissue to achieve approximate correlations between susceptibility-weighted imaging (SWI) and iron maps for multiple anatomical regions in each case via the rapid SXRF approach (Hopp et al., 2010), and for the first time in unfixed tissues, working at high spatial resolution in thin sections to match SXRF iron maps with R_2_ and R_2_* maps, permitting detailed analysis of the hippocampus in AD cases and age-matched controls and demonstration of a positive correlation between iron and R_2_, R_2_* (Antharam et al., 2012).

### INVESTIGATING EVIDENCE OF OXIDATIVE STRESS IN NEURODEGENERATIVE DISEASE WITH SXRF

The role of iron in neurodegenerative disorders is often considered in the framework of oxidative stress (Kell, 2010; Smith et al., 2010). Analysis of selenium is important in this context, as it is an essential element found in the cofactor for glutathione peroxidase, which is involved in regulating oxygen free radicals. Selenium is likely very important in protecting against free radical damage from poorly liganded iron in neurodegenerative disorders. SXRF is especially useful for sensitive and specific analysis of selenium in tissue (Schulmann-Choron et al., 2000), where Schulmann-Choron and colleagues obtained SXRF spectra from acid-digested rat brain tissue, and reported a minimum detection limit (MDL) of 20 ppb for selenium, using the full beam (focused to approximately 1 mm^2^), and counting for 500 s per acquisition. Ince et al. (1994) had reported elevated iron and selenium in lumbar spinal cord from MND cases compared with controls using NAA, and in spatial SXRF analysis of SN and spinal cord in PD and ALS cases, selenium was selectively observed in the neuronal bodies of the SN (Szczerbowska-Boruchowska et al., 2004). Subsequently, the SXRF investigation of neuromelanin in normal SN led to the observation that selenium concentration in neuromelanin appears to increase with age, and may indicate increased requirements for protection against oxidative stress as a function of aging (Bohic et al., 2008).

## XAS: LOOKING AT THE OXIDATION STATE OF IRON AND ITS LOCAL ENVIRONMENT

Determining absolute concentrations of tissue iron, and the proportion of iron to which MRI sequences are sensitive, is important to help with recognizing disruptions to iron homeostasis and to identify potential clinical markers of disease. However, the questions can become more subtle when we consider underlying mechanisms of iron-mediated toxicity. Some instances of tissue iron overload may be a consequence of impaired bioavailability (Rouault, 2013; Visanji et al., 2013), and under certain circumstances the colocalization of iron and species with, for example, reductase potential like Aβ_42_ (Khan et al., 2006) or α-synuclein (Davies et al., 2011), may be a more significant contributing factor to oxidative stress damage than tissue iron concentration in its own right (Gallagher et al., 2012; Everett et al., 2014a). As SXRF may be used non-destructively, there is plenty of scope to correlate SXRF iron maps with other parameters relevant to iron-mediated toxicity, and to probe the oxidation state and mineral form of iron within SXRF maps by techniques such as XANES (Collingwood et al., 2005a; Bacquart et al., 2007).

Two decades ago, De Stasio et al. (1995) used X-ray secondary-emission microscopy (XSEM) to spatially evaluate the chemical distribution of iron and other metals in isolated rat cerebellar granule cells after they had been exposed to iron in solution. The iron absorption spectrum was measured between 50 and 60 eV, and a difference image at the iron absorption edge between the 55 and 54 eV energies was calculated to gain a map of iron distribution. The spatial distribution of the elemental analysis was 0.5 μm or better. This particular experiment, perhaps due to some limitation in the protocol, did not enable the association of iron with particular cells or cellular structures to be identified; rather, iron was found throughout the specimens. However, in principle the absorption profile can contain a great deal of information about the local chemical environment of the absorbing species, and below we consider how XAS, specifically EXAFS and XANES, have been applied to the study of iron in the brain.

Ascone and Strange have reviewed in detail the contribution of XAS to the analysis of metalloproteins (Ascone and Strange, 2009), using the descriptor “*metalloproteomics*” defined as the “*structural and functional characterization of metal-binding proteins.*” They acknowledge that XAS in biological materials requires a synchrotron X-ray source to achieve the necessary flux, stability of the beam and associated optics, and the ultra-sensitive fluorescence detectors, along with a variety of highly regulated sample environments. Concerning XANES of biological materials, or “bio-XANES,” the importance of the pre-edge spectrum, the developments in gaining quantiative structural information from bio-XANES, and some limitations of “fingerprinting” with XANES are discussed. XANES and EXAFS continue to be excellent tools for analysis of the electronic structure for metal elements in biological materials, and with an expanding cross-disciplinary user base, there have been improvements in both the sophistication and accessibility of purpose-designed (and open source) software tools for analysis of the spectra (Ascone and Strange, 2009). More complete studies can be undertaken by combining synchrotron methods such as EXAFS and X-ray diffraction for structural determination of iron sites in multi-metal-bearing proteins (Einsle et al., 2007) for metalloproteins such as ceruloplasmin (Ascone and Strange, 2009), or to evaluate trafficking of metal ions between proteins. Improvements in beamline instrumentation continue to improve technical scope and measurement efficiency, and rapid acqusition of high-quality XANES has enabled XANES “mapping” at some beamlines with XANES obtained at each pixel in the sample map.

### X-RAY ABSORPTION SPECTROSCOPY TO STUDY IRON AND NEUROMELANIN IN THE SUBSTANTIA NIGRA

Many experiments have now been performed to compare iron in the SN in the healthy and PD brain. These experiments have primarily been used to image or probe the distribution of iron in tissue sections by various qualitative and quantitative methods, or iron concentrations have been determined for bulk samples. Spatial analysis of iron in unfixed tissues of the SN has shown some evidence for site-specific elevation of iron in PD dopaminergic neurons compared to healthy controls (Szczerbowska-Boruchowska et al., 2004; Oakley et al., 2007), but elevated iron concentration alone does not provide information about the chemical form of the iron, or indicate whether excess iron is bound in a state that differs from the iron normally present. Here, we review XAS studies that have attempted to address this question in the context of PD.

The dopaminergic neurons of the SN are selectively vulnerable in PD and confirmed to accumulate iron in the disease state (Dexter et al., 1989; Oakley et al., 2007; Crichton et al., 2011). These neurons are normally pigmented with neuromelanin, which has a strong affinity for metal ions including iron (Double et al., 2003). X-ray microanalysis was performed on an electron microscope to determine Fe^2+^ and Fe^3+^ associated with various forms of neuromelanin, and from this it was deduced that the primary form of iron in dopaminergic neurons is neuromelanin-bound iron, and that neuromelanin preferentially binds Fe^3+^ (Jellinger et al., 1992). X-ray analysis at various synchrotron facilities has subsequently been used to further explore the nature of iron storage in neuromelanin, which is considered important to understand if we are to fully explain the contribution of iron-mediated toxicity in PD.

**FIGURE 3:**
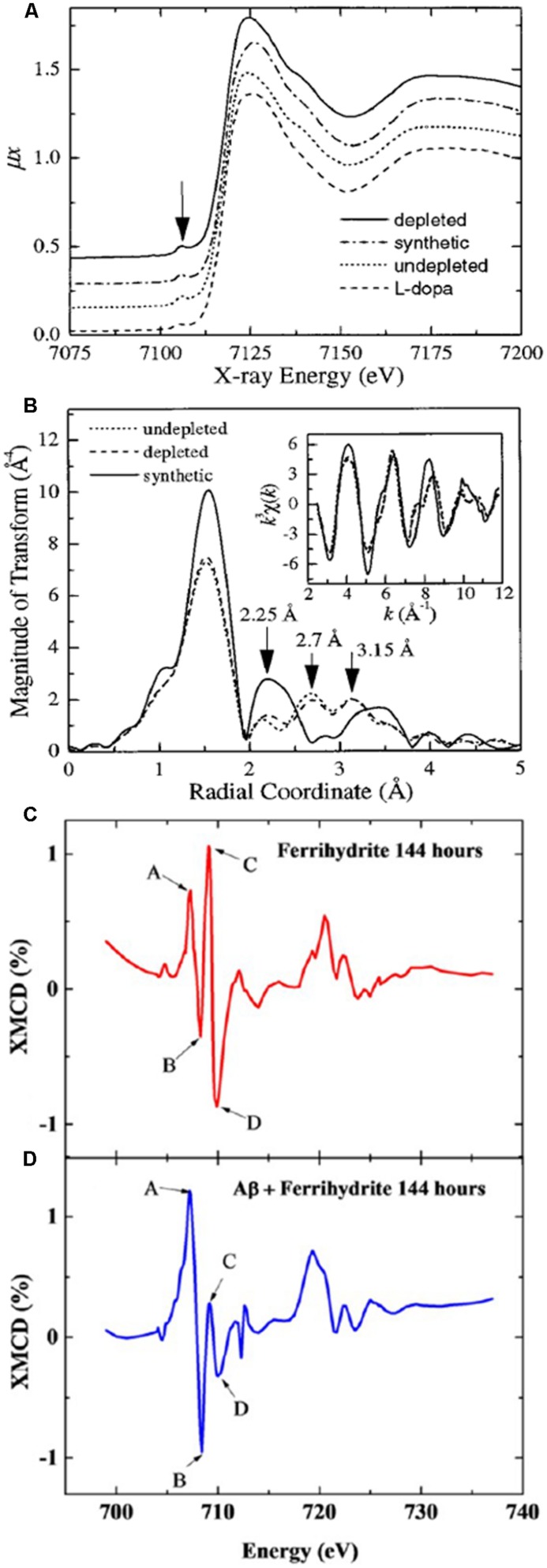
**X-ray absorption spectroscopy analysis of iron**. Panel **(A)** shows EXAFS spectra of iron bound to synthetic, natural 100% saturated, and natural 30% saturated (depleted) neuromelanin, where the traces appear similar. Panel **(B)** shows the processed EXAFS data reveal marked differences between the synthetic and natural neuromelanin. Panels **(C**,**D)** show XMCD spectra collected after 144 h for **(C)** ferrihydrite, and **(D)** ferrihydrite incubated with Aβ_42_, where the positive and negative peaks (A,B,C,D) indicate an antiferromagnetic structure, and the dramatically increased amplitude of peaks (A,B) in panel **(D)** is consistent with a significant increase in the proportion of Fe^2+^ in the antiferromagnetically ordered mineral. Panels **(A,B)** were published in Kropf et al. (1998), Copyright Elsevier (1998). Panels **(C,D)** are adapted with permission from Everett et al. (2014a). Copyright 2014 American Chemical Society.

Kropf et al. (1998) used EXAFS to look at the structure of human neuromelanin and its analogs. They observed that both human and synthetic neuromelanin have a common iron center within a sixfold distorted oxygen octahedron, with a distance ∼ 2 Å. Earlier studies with Mössbauer spectroscopy had reported evidence for superparamagnetic behavior in both synthetic neuromelanin and purified human neuromelanin from the SN; in the latter study, the parameters were observed to be similar to those found for hemosiderin and ferritin (Gerlach et al., 1995). Kropf et al. (1998) investigated the possibility that there are two binding sites in neuromelanin, by looking with EXAFS at the near-neighborhood environment of iron in both extracted human and synthetic neuromelanin. The study included samples that were either fully or partially (30%) saturated with iron, where for the latter the most easily chelatable iron had been removed. As the partially saturated human neuromelanin spectrum was virtually indistinguishable from the saturated version, it was concluded that there were no differences in the affinities of the binding sites (**Figure 3**). Melanin aggregation had formerly been described using a fractal construct, implying a sponge-like structure, and Kropf and colleagues reported their EXAFS observations as being consistent with this model, suggesting that instead of two iron binding sites, iron would simply be removed more rapidly from outer than inner sites for a neuromelanin particle. The structural information from the EXAFS indicated that a superparamagnetic structure would be unlikely. Mössbauer spectroscopy was then used to demonstrate that the neuromelanin exhibited only paramagnetic behavior (Kropf et al., 1998), and it was concluded that the iron in neuromelanin does not follow the ferritin model in this regard.

In a parallel study, EXAFS was used to analyze iron in whole tissue samples that had been gently homogenized prior to analysis (Griffiths et al., 1999). The study included samples from the SN and GP regions in a group of PD cases and healthy control brains, with *n* = 6 in each group. For the tissue, it was concluded that ferritin-like parameters provided the most appropriate fit for both anatomical regions. This result for SN is not necessarily in contradiction of the former neuromelanin analysis (Kropf et al., 1998), which was performed on isolated neuromelanin and would not have included iron from other sources such as astrocytes, oligodendrocytes, and activated microglia in the vicinity of extracellular neuromelanin. Indeed, it has been suggested that neuromelanin-bound iron would not exceed ∼15% iron in the SN (Galazka-Friedman et al., 1996). Given the heterogeneous nature of iron and neuromelanin distribution throughout the subfields and cell types in the SN (Morris and Edwardson, 1994), the exact region dissected for analysis will influence the proportion of tissue iron that is neuromelanin-bound; it has also been suggested that the strong affinity of neuromelanin for iron leads to additional iron from other sources (such as glia) binding neuromelanin during the extraction and purification process, as neuromelanin is not normally saturated (Double et al., 2003). Based on the number of parameters required to fit clusters of electron-dense cores in these same samples (observed by electron microscopy), it was concluded that a greater degree of sub-cellular clustering occurred within PD tissue than in controls (Griffiths et al., 1999). While this was interpreted as a change in sub-cellular clustering of ferritin, it is possible that for SN this observation included neuromelanin clusters, consistent with former histopathological observations and a concurrent study from Ektessabi et al. (1999) using SXRF imaging of PD and control SN where they noted “*various sizes of melanin granules released from dying nigral neurons scattered in a more condensed form (neuromelanin aggregates) than those in the nigral neurons of the control subject.*”

Ektessabi et al. (1999) had originally been unable to obtain useful XANES data from the formalin-fixed SN tissues that they mapped by SXRF, but at the 39XU Spring-8 Japanese synchrotron they mapped a small area at 7.2 keV, just above the iron K-edge, prior to obtaining XANES in the fluorescence configuration with a beam spot limited with a pinhole to approximately 10 μm in the sample plane (Yoshida et al., 2001). Reference standards of FeO and Fe_2_O_3_ were used in order to determine the position of the Fe^2+^ and Fe^3+^ edges, respectively, and to thereby compare relative proportions of Fe^2+^ and Fe^3+^ at sites of interest throughout the SN. It is reported that with a 0.5 eV energy step, that dwell times ranged from 20 to 100 s per step (Yoshida et al., 2001). While comparisons between neuromelanin-bound iron in intact and dead neurons were made (which suggested an increased fraction of Fe^3+^ in neuromelanin associated with degenerating neurons), the use of a single case, and the prior fixation and paraffin embedding of the tissue means that this study is more significant as a demonstration of potential for iron XANES from individual cells in sections of human brain tissue.

MicroXANES in unfixed SN tissue was performed by Chwiej et al. (2007), where a single PD case and six controls were compared in freeze-dried tissue. Regions of pigmented neuromelanin, consistent with those observed in dopaminergic neurons, were selected for XANES analysis, and no significant difference between the PD and control samples was detected. In contrast with the prior XANES reported from fixed tissue (Yoshida et al., 2001), the only detectable chemical state of the iron in these unfixed tissues was Fe^3+^ (Chwiej et al., 2007). This observation, while it contradicts prior reports of Fe^2+^ in normal PD SN, is strongly supported by an earlier Mössbauer analysis of SN from PD and control cases, where only Fe^3+^ was detected in bulk tissue samples (Galazka-Friedman et al., 1996).

The XANES study by Baquart el al, analysing PC12 cells as a model system for the SN (Bacquart et al., 2007), is especially thorough in assessing the impact of sample preparation and beam exposure on the scope for radiation damage in the form of mass loss (Williams et al., 1993) and photoreduction (Yano et al., 2005). This XANES study was concurrent with members from the same team reporting high resolution SXRF analysis of iron and dopamine vesicles in the same cell model (Ortega et al., 2007). XANES was selected for its scope for measurement with minimal preparation of the cells, avoiding processes that might modify the chemical species. Beam focus of 1.5 μm × 4 μm was achieved with K–B mirrors, and iron and arsenic were analyzed by XANES captured in fluorescence mode (which is optimal for dilute specimens). Regions sampled within the PC12 cells included cytosol, the mitochondrial network, and nucleus. It was observed that cells measured in the frozen hydrated state (maintained at –100^∘^C, in a liquid nitrogen cryostream) were generally preferable to freeze-dried cells at room temperature, as this increased repeatability, sensitivity, led to no noticable shift in oxidation state, and minimized beam damage. Interestingly, however, it was observed that for iron, any change in chemical state between the frozen hydrated and freeze dried cells was insignificant in the XANES, and that the technically more straightforward method of working with the freeze dried cells at room temperature, facilitating observation with the videomicroscope during measurement, made this the preferred option (Bacquart et al., 2007). To achieve good signal to noise, with minimal beam damage to the sample, it was noted that short repeated energy scans were preferable to a single scan with longer dwell times at each energy. Iron XANES at the K-edge (7.112 keV) were obtained over the energy range 7.037–7.200 keV, collecting for 5 s/step, with a step size of 1.0 eV pre- and post-edge, and 0.5 eV through the edge region (7.082–7.142 keV), giving a scan time of approximately 18 min. (Scan times longer than this led to significant signal decay for XANES of arsenic in the freeze dried cells at room temperature, indicating mass loss of this element. Mass loss was tracked by decreasing white line intensity; notably, the dose observed to cause mass loss in the hard X-ray region at room temperature did not have the same effect with soft X-rays at 113 eV, or under cryoconditions, where both protected against mass loss. Minimizing the dose was also important to reduce scope for photoreduction by the X-rays.) The microfocussed achromatic beam was very stable; beam movement on the sample corresponded to a displacement of approximately 0.1 μm over 100 eV. While the short XANES scans could be repeated and summed to improve signal quality without causing detectable sample damage, the iron concentration in the PC12 cells was sufficient that good XANES data were acquired in a single acquisition. The “limit of speciation” for elements in the atomic mass range of 20–40 was judged to be approximately 13 μg/g (Bacquart et al., 2007). Progress in synchrotron technology is resulting in samples being exposed to higher photon flux, so the conditions required for sample preservation will require ongoing evaluation (George et al., 2012).

### X-RAY ABSORPTION SPECTROSCOPY TO STUDY IRON IN FRIEDREICH’S ATAXIA

In order to study mitochondrial iron chemistry in the context of Friedreich’s Ataxia, Popescu et al. (2007) analyzed the heavy mitochondrial fraction isolated from primary fibroblasts taken from individuals with and without Friedreich’s Ataxia, and analyzed them by XANES at the iron K-edge. In order to determine the mineral state of the iron, they fitted the spectra with a library of 22 iron compounds, and concluded from the fitting results (with supporting data from Western blotting) that the mineralized iron in the Friedreich’s Ataxia patients is more highly organized than in the unaffected individuals, and that the Friedreich’s Ataxia patients mineralize a significant fraction of cellular iron in mitochondrial ferritin (MtFt; Popescu et al., 2007). MtFt has been postulated as having a protective role for mitochondria in cells which have high levels of metabolic activity and oxygen consumption, rather than being associated with iron storage (Levi et al., 2001), and since Popescu’s XANES analysis, this potentially protective role has been explored specifically in the context of Friedreich’s Ataxia (Campanella et al., 2009).

## COMBINING SYNCHROTRON X-RAY FLUORESCENCE MAPPING AND ABSORPTION SPECTROSCOPY

As the microfocus SXRF and XANES techniques mature, there has been increased recognition of their complementarity, and many microfocus beamlines are now well-equipped to measure both during the course of an experiment (**Figure 1**). Practical measures such as simultaneous acquisition of CCD data in transmission mode to determine crystalline parameters of biominerals, and introducing custom lithographic finder grids to aid location and retrospective analysis of sites within a sample, have previously been proposed (Collingwood et al., 2005b).

### INVESTIGATING IRON BIOMINERALIZATION IN ALZHEIMER’S DISEASE

One of the early applications of the combined SXRF and XANES analysis was to demonstrate how isolated microscale iron focii in large (≥1 cm^2^) areas of tissue could be efficiently identified and characterized in a way that is impractical or impossible with other microprobe techniques. The motivation for this work was the detection of tiny deposits of magnetite, a mixed valence iron oxide, in human brain tissue, via analysis of bulk tissue samples and isolated material (Kirschvink et al., 1992; Dunn et al., 1995). There were early indications that levels of magnetite were elevated in AD tissue compared for healthy controls (Hautot et al., 2003), although the extent of this, and the mechanism for its formation *in vivo*, was relatively unexplored. Our preliminary work with avian tissue (Mikhaylova et al., 2005), known to contain particulate magnetite, established the protocols for the first demonstration of magnetite *in situ* in human brain, using AD amyloid-plaque-rich tissue from the superior frontal gyrus (Collingwood et al., 2005a). SXRF mapping at resolutions ranging from 100 to 5 μm were used to detect and precisely locate iron focii, and XANES analysis was performed with the 5 μm beam spot to determine the forms of iron present at the sites, which primarily included ferritin-like ferrihydrite, and/or magnetite. The combination of SXRF and XANES has since been used to study the amyloid-β precursor protein (AβPP)/presenilin 1 (PS1) mouse model of AD (Gallagher et al., 2012). This model is engineered to over-express the Swedish mutation of human AβPP, and mutant human PS1, and is known to exhibit amyloid deposition in the brain from approximately 6 months. SXRF and XANES were used to demonstrate magnetite deposits in amyloid-rich regions of the AβPP/PS1 transgenic mouse brain (Gallagher et al., 2012), and in this study we suggested that the magnetite deposits are an indication that iron dysregulation is an early event in AD-related pathology. Subsequently, in work led by the Telling group, we suggest a mechanism, demonstrated *in vitro* by synchrotron methods including XAS and X-ray magnetic dichroism (XMCD), by which the reductase behavior of Aβ_42_ may lead to the observation of magnetite in these plaque-rich tissues (Everett et al., 2014a).

### UNDERSTANDING NEUROMELANIN

Another area in which the combination of SXRF and XANES has been utilized, is in the study of neuromelanin in human tissues (Bohic et al., 2008). SN tissue was obtained from formalin-fixed paraffin embedded samples from seven human brains ranging in age from 24 weeks to late adulthood, and the cases were selected on the basis of having no significant neuropathology, SN related pathology, or dopamine-related disorders. Pigmented neurons were SXRF mapped, and then XANES obtained at the K-edge in fluorescence mode in 1 eV increments over the range 7.07–7.37 keV. The authors report increased levels of cellular neuromelanin, followed by a darkening of the pigment, as a function of aging. From the trace metal mapping it was established at a high level of spatial resolution that iron and selenium are closely associated with neuromelanin from an early stage of development, and that other metals such as calcium, copper, and zinc become associated with it at a later stage. The levels of neuromelanin-bound iron in the tissue were below the expected saturation value for neuromelanin, consistent with prior work (Double et al., 2003). The intensity of the SXRF spectra indicate that neuromelanin-bound iron increases as a function of age, although the microXANES spectra were consistent with ferritin regardless of age. It should be noted that the chemical form of iron in the SN was shown by Mössbauer to be demonstrably different in formalin-fixed archive samples compared for fresh-frozen (Galazka-Friedman et al., 1996), but here the XANES conclusions are generally consistent with those previously obtained in unfixed sections (Chwiej et al., 2007). Bohic et al. (2008) conclude, contrary to prior EXAFS observations in extracted human neuromelanin (Kropf et al., 1998), that neuromelanin is likely to have variability in its metal binding domains that suggest different functional roles. There is, however, unanimous agreement between all four studies cited here that the iron bound to neuromelanin is in the Fe^3+^ form within the limits of experimental error. A subsequent study of extracted neuromelanin granules was conducted by Tribl et al. (2009), where a variety of proteins were shown to be associated with neuromelanin, including L-ferritin. This observation may in due course contribute to explaining the multiple binding affinities reported for neuromelanin, and arguably there is an opportunity here to combine immunohistochemistry with sub-micron SXRF and XANES to determine if and how L-ferritin colocalises with neuromelanin in intact pigmented cells.

## IRON-BINDING PROTEINS AND AGGREGATION

The main emphasis of the previous sections in this review has been on the use of synchrotron techniques to locate and characterize iron, including that which is tightly coordinated to metalloproteins. Synchrotron analysis also has the scope to provide insights where metalloprotein structure and conformation is concerned, which is an essential aspect of understanding iron regulation (and by extension dysregulation) in neurodegenerative disorders.

Synchrotron techniques have been used for analysis of a variety of iron-binding metalloproteins, including transferrin, hemoglobin, and ferritin, where the latter has been investigated in many synchrotron experiments to further understand both the protein structure, and the nature of the iron oxide core formed within (Kim et al., 2011). The powerful combination of high resolution X-ray crystallography and EXAFS has been especially useful in determining the structure of metalloproteins (Hasnain, 2004; Duke and Johnson, 2010). One of the advantages of synchrotron X-ray crystallography is the rapidity with which diffraction patterns can be obtained from highly complex structures. Several groups have used time-resolved Laue diffraction studies to investigate structure and conformational properties of metalloproteins (Duke and Johnson, 2010). For example, Bourgeois et al. (2003) reported the application of nanosecond Laue crystallography to determine protein structure using myoglobin as model system. This study with 150 ps X-ray pulses enabled room temperature observation of the dynamics of protein conformational changes.

### SMALL ANGLE X-RAY SCATTERING

A widely used synchrotron technique for the conformational study of metalloproteins is small angle X-ray scattering (SAXS), which has been used to look at proteins intimately involved in the uptake and transport of iron. As with crystallography, time resolution can be a useful feature in SAXS measurements, enabling transient aggregates to be distinguished from folding intermediates (Segel et al., 1999). Castellano et al. (1993) used SAXS to look at the conformational changes in aggregates of human transferrin. They looked specifically at transferrin from human serum (serotransferrin), an 80 kDa metal binding protein which is structurally modified on binding to metal ions, and which has sites for two Fe ions. This investigation was motivated by a desire to understand the potential role of transferrin in binding Cu and Al in addition to Fe. Castellano’s study included observations of apotransferrin and monoferric transferrin, and they concluded that the conformational changes brought about by the iron binding process were consistent with prior observations made with both X-ray and neutron scattering methods. The transferrin was considered in a fractal framework, permitting particle and cluster sizes to be distinguished (Castellano et al., 1993). More recently, it has been observed that transferrin can form fibrils under certain conditions *in vitro*, and this led to an investigation to determine whether transferrin has amyloid-like properties. Synchrotron X-ray circular dichroism (XCD) was used to test whether transferrin in solution has amyloid-like properties, and this helped show that transferrin aggregation in solution does not appear to involve major structural changes to the protein, or formation of beta-pleated sheets (Booyjzsen et al., 2012).

Small angle X-ray scattering is ideal for studying protein (mis)folding and aggregation problems, which are a common feature in neurodegenerative disorders. In turn, iron and other metal elements are associated with modifying or exacerbating protein aggregation in the majority, if not all, of these disorders (Kell, 2010). SAXS permits the size and shape of soluble aggregates to be characterized, and can provide information about the order of aggregation (i.e., whether it is a dimer, trimer, or higher order). One protein that has been studied in detail by SAXS is α-synuclein, which forms insoluble fibrils in neurodegenerative disorders classed as the “synucleinopathies,” including PD, dementia with Lewy bodies (DLB), and MSA. Uversky et al. (2002, 2005) conducted a series of studies incorporating SAXS, which investigated the conformation, shape, and association of the α-synucleins in solution. Typical experiments were performed at room temperature, and involved the α-synuclein passing through a flow cell along a 1.3 mm path, with 25 μm mica windows (Li et al., 2001). SAXS permitted wild-type, and familial PD point mutations (A30P and A53T) to be distinguished, determining the radius of gyration, *R*_g_, and the confirmation and globularity (indicating packing density) of the protein. It was observed that the wild-type and mutated forms of α-synuclein have identical *R*_g_ of 40 Å at neutral pH, and that this value decreases as the pH is lowered, indicating compacting of the protein giving rise to a reduction in volume. The homogeneity of the proteins, the absence of aggregation under these conditions, and the configuration approximating to a random coil at neutral pH is also demonstrated through the SAXS data (Li et al., 2001). Subsequent studies considered in more detail the monomeric and fibrillar forms of α-synuclein, including exploring factors that promote and inhibit aggregation (Uversky et al., 2002, 2005). Recent advances in time-resolved XAS include work from Lima et al. (2011) to enable MHz-rate data acquisition with picosecond lasers to enable studies at physiologically relevant concentrations for biological systems.

### SYNCHROTRON FOURIER-TRANSFORM INFRA-RED ANALYSIS

To study proteins such as α-synuclein, Aβ, and prion protein in intact tissues, another technique may be used: FTIR spectroscopy, where the infrared spectrum is obtained from a given material. FTIR is routinely performed using laboratory sources, but for certain measurement configurations, synchrotron light sources have capacity to achieve brightness that is orders of magnitude brighter than standard sources. A detailed review of the principles of the instrumentation, and of approaches to biological specimen preparation for FTIR, is provided by Miller and Dumas (2006), who consider FTIR micospectroscopy (FTIRM), and imaging (FTIRI). Where the beam spot size at the sample is determined by a pinhole, synchrotron FTIR is especially advantageous for microscale analysis with resolution ∼ 10 μm. The spatial resolution of FTIRM is limited by the wavelength of the infrared, from approproximately 1.7 μm at 4000 cm^-1^ to 13 μm at 500 cm^-1^. Spectra from biological samples are obtained in transmission mode with thin samples (typically 5–30 μm), or in reflection mode for highly reflective or unsectionable samples (Miller and Dumas, 2006).

Fourier transform infrared may be used to study protein folding dynamics on the microsecond time scale, at good spatial resolution and with a small volume of sample. While slower than laser techniques, the white beam ensures the complete spectra can be obtained (Miller and Dumas, 2006). However, one of the most interesting applications of FTIR is the subcellular chemical mapping that can be achieved, which is highly complementary to the analysis of metal and mineral structures outlined previously in this review. FTIR gives insight into the nucleic acid, protein, and lipid content of particular structures, and does not significantly heat the sample, so that prolonged studies of individual living cells may be performed. Multimodal imaging, such as combining SXRF and FTIRM with epifluorescence microscopy to look at trace metals and AD senile plaques (Miller et al., 2006), can be done with fluorescent tag concentrations at levels that do not interfere with the infrared, and there is growing interest in combining synchrotron FTIR, SXRF, and XAS (Miller et al., 2006; Kastyak et al., 2010). For example, Kastyak et al. (2010) used synchrotron FTIR to analyze crystalline deposits of creatine detected in SXRF-mapped unfixed tissue from spinal cord, brain stem, and motor neuron cortex in cases of ALS. Intense focii (“hot spots”) of calcium, zinc, iron, and copper were also noted in the ALS tissues. Miller et al. (2006) initially demonstrated the combination of SXRF microfocus mapping and FTIR to unambiguously associate iron, copper, and zinc with Aβ deposits in unfixed air-dried human brain tissue from AD cases. FTIRM and SXRF were mapped with a resolution of 5–10 μm, and the Amide I absorbance was used to confirm the presence of Aβ. Tissue sections were mounted on quartz after the slides had been coated with a 200 nm sputtered coating of aluminum. The latter was necessary for infrared reflectivity, but the spatially heterogeneous trace contamination of iron in even this thin aluminum layer place some constraints on the tissue iron analysis that could be performed in this study. The copper and zinc distributions (as demonstrated by SXRF) showed good correlation with regions containing Aβ deposits (as demonstrated by FTIR). In a subsequent study, using Ultralene^®^ as an ultra-clean SXRF and FTIR compatible substrate for the tissue samples, the correlation between transition metal ions and amyloid deposition was investigated for the AβPP/PS1 mouse model of AD (Leskovjan et al., 2011). Sections were cut at 30 μm thickness, air-dried, and kept in a dessicator prior to imaging. Approximate quantification of elemental concentrations was achieved using thin film standard reference materials. Amyloid was visualized with a simple Thioflavin-S staining protocol, where they observed no change in the iron, copper, or zinc content of the map subsequent to staining, and FTIRM was used (in transmission mode) to determine tissue protein density using a spatial mapping resolution of 4 μm. Normalization of metal ion content to protein content for each selected plaque was used to determine whether iron, copper, and zinc accumulate in large dense areas of amyloid deposition in the AβPP/PS1 model of AD; it should be noted that only large plaques (30–50 μm) were included in the quantitative correlations, to ensure they occupied the full thickness of the section. Analysis of regional iron, copper, and zinc distribution was achieved by a combination of standard segmentation (e.g., of the hippocampus) and by cluster analysis. Interestingly, with SXRF microscopy, an elevation (∼1/3) of cortical iron was observed in the AD model compared to the wild type, which is consistent with observations from Smith et al. (2010) in human AD and the iron was not directly associated with the large regions of amyloid deposition that were included in the analysis. Zinc, however, became associated with the amyloid deposits at the end-stage of the study (56 weeks), having not demonstrated elevation at 24 and 40 weeks. Leskovjan et al. (2011) acknowledge that there may possibly be a relationship between brain iron and the amyloid pathology, but given that iron was not specifically elevated at the amyloid-plaque sites, when normalized to protein content, there was clearly some doubt. Their study highlights an important question about the manifestation of iron dysregulation in AD: does tissue iron have to be significantly elevated above normal concentrations in order for iron to contribute to pathophysiological processes?

## DISCUSSION

### DOES IRON-MEDIATED TOXICITY DEPEND ON IRON CONCENTRATION?

Historically, attention has been paid to brain cells, tissues, and even whole brain regions exhibiting marked changes in iron concentration. These indicate candidate sites vulnerable to iron-mediated toxicity, but analyses that focus purely on iron concentration in tissue have been challenged (Friedman et al., 2009), and lead in many cases to conflicting observations (Friedman et al., 2009; Schrag et al., 2011), and the contribution of iron overload to pathology is unclear (Rouault, 2013). Kell considers the problem from the perspective of poorly liganded iron (Kell, 2010), placing emphasis on the local chemical environment, and the availability of iron to participate in reactions that stimulate the overproduction of damaging radical species. We suggest here that significantly elevated concentrations of iron are not a prerequisit for this scenario.

We will take Aβ aggregation as a case study, where the interactions between Aβ_42_ and iron *in vitro*, and the relationship between Aβ and iron deposition in humans and AβPP mouse models, have been characterized by synchrotron methods (Collingwood et al., 2005a; Gallagher et al., 2012; Everett et al., 2014b). Many studies have demonstrated that iron is associated with insoluble deposits of Aβ in human brain, even in the pre-clinical stage of AD (Smith et al., 2010), and it is understood that iron provides much of the natural contrast that makes amyloid plaques observable in high resolution MRI (Meadowcroft et al., 2009; Petiet et al., 2011). However, observed accumulation of iron in amyloid plaques is reportedly lower in mouse models of AD than in human tissue (Leskovjan et al., 2009; Meadowcroft et al., 2009), and tissue iron concentration has been shown not to correlate with plaque burden in human AD cases (House et al., 2008). This observation was reinforced in study of 60 aged human brains, where it was demonstrated that there is no relationship between tissue iron concentration and congophilic amyloid angiopathy or senile plaque burden (Exley et al., 2012). The formation of spherulites from Aβ_42_ has been demonstrated in the presence of copper (House et al., 2009), and it is postulated that these structures, which form *in vitro* and are also observed in human brain tissue, may correspond to the senile plaques with fibrillar structure routinely observed with transmission electron microscopy. SXRF microfocus analysis of the spherulites in unfixed human AD hippocampus revealed direct association of the spherulites with copper, not iron, despite iron being abundant in the tissue (Exley et al., 2010). Subsequent Perls staining in formalin-fixed human hippocampus from another individual also showed no direct positive correlation between iron and spherulite distribution (House et al., 2011). In our SXRF analysis of AD and control hippocampus, where microfocus-resolution iron maps were correlated with MRI microscopy, again no disease-dependent change in total regional iron level was observed, although the AD tissue exhibited a higher proportion of R_2_ and R_2_* hyperintensity artifacts consistent with iron-rich deposits within the hippocampus (Antharam et al., 2012). It is therefore necessary to consider not only concentration of iron and its co-localization (or otherwise) with Aβ, but also the chemical and bound state of iron in tissues exhibiting amyloid aggregation. Synchrotron facilities provide exceptionally sensitive and unambiguous techniques for this purpose. Our study with SXRF and XANES to investigate the mineral form of iron deposits in human AD plaque-rich cortical tissue demonstrated the presence of ferrihydrite (Fe^3+^, consistent with normal ferritin cores) and magnetite (alternating lattices of Fe^2+^ and Fe^3+^) within the tissue (Collingwood et al., 2005a); an observation subsequently supported by the detailed characterization of ferritin-core-sized magnetite/maghemite iron oxide particulates in extracted amyloid plaque core materials (Collingwood et al., 2008b). The subsequent SXRF and XANES analysis in the AβPP/PS1 model produced essentially the same observation concerning magnetite formation, despite quantitative analysis of the same brains showing no overall difference in brain iron concentration between wild-type and the AD mouse model (Gallagher et al., 2012).

**FIGURE 4:**
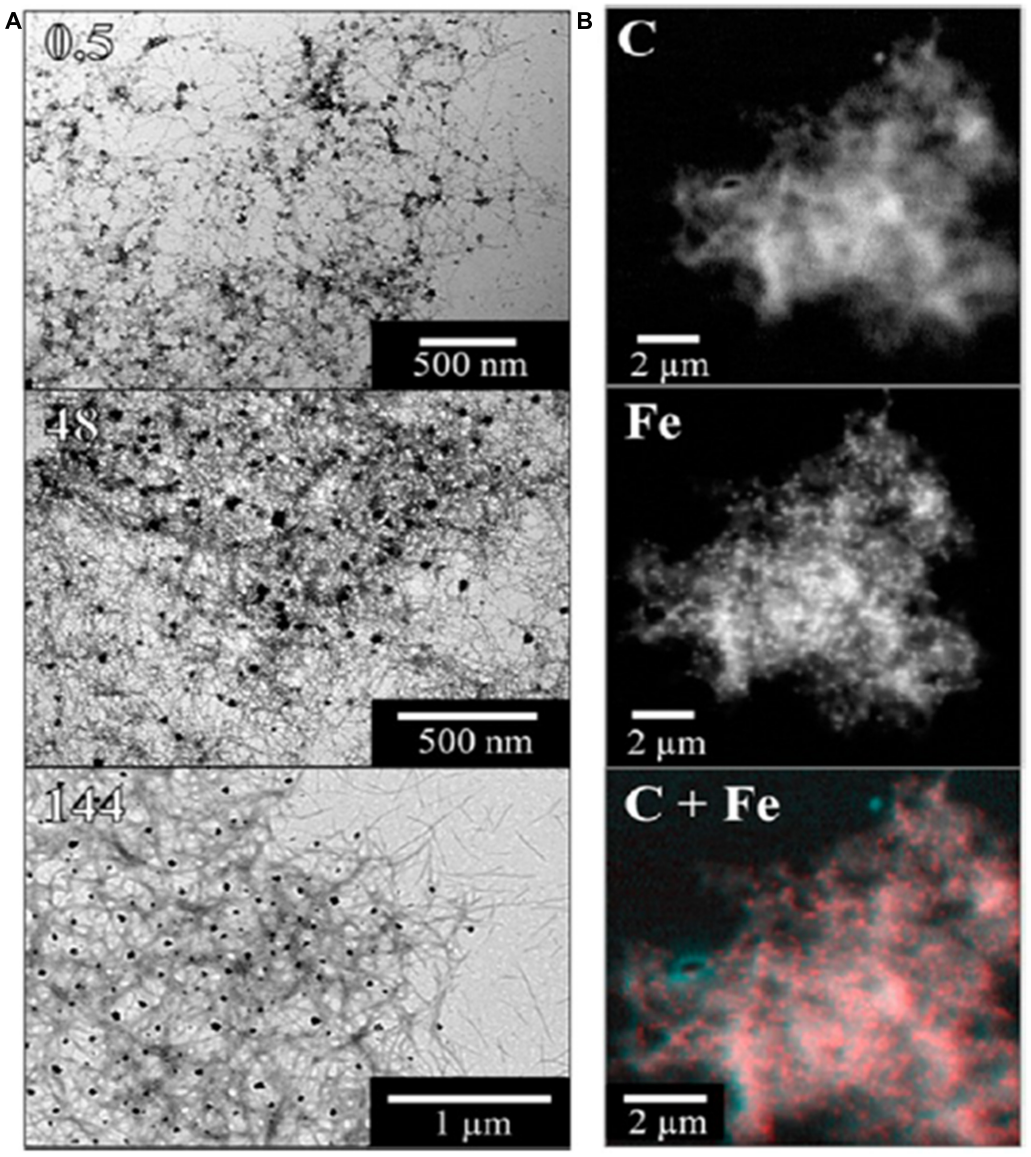
**Illustration of STXM chemical mapping of iron associated with aggregated Aβ_42_.** Panel** (A)** shows bright-field transmission electron microscopy (TEM) images and panel **(B)** shows scanning transmission X-ray microscopy (STXM) images of fibrillar amyloid structures formed following the incubation of Aβ_42_ with ferrihydrite. TEM pictures show Aβ structures present after 0.5, 48, and 144 h of incubation. STXM images show the carbon (C) and iron (Fe) content of an Aβ/ferrihydrite aggregate, along with a carbon/iron (C + Fe) composite image of the same aggregate. Reprinted (adapted) with permission from Everett et al. (2014a). Copyright 2014 American Chemical Society.

What is the origin of the mixed valence oxides observed in the human and mouse model tissues, and is their association with regions exhibiting Aβ deposition a coincidence? The reductase behavior of Aβ_42_ peptide, a primary component of amyloid pathology in AD, has been demonstrated *in vitro*, where it has the capacity to reduce iron from its more stable ferric (Fe^3+^) form to the more reactive bio-available ferrous (Fe^2+^) form at physiological pH (Khan et al., 2006; Everett et al., 2014b). Using a combination of XAS (using the total electron yield method), and XMCD by obtaining two XAS spectra with opposed magnetic fields oriented in the direction of the X-ray beam (**Figure 3**), we have now shown that Aβ_42_ has the capacity to chemically reduce iron in ferrihydrite particles (Everett et al., 2014a), and the results from this *in vitro* study support the possibility that the Fe^2+^ oxide that forms in the presence of Aβ_1-42_ is a precursor to magnetite formation (Everett et al., 2014a). These results are further supported by chemical imaging using STXM (**Figure 4**). Cellular ferrireductase behavior by α-synuclein, a peptide which is a key constituent of protein aggregates in PD, DLB, and MSA, has also been observed (Davies et al., 2011). These results indicate that co-localization of trace levels of labile or poorly liganded iron with aggregating Aβ_1-42_ or α-synuclein has the potential to lead to significant overproduction of radical species in various neurodegenerative disorders.

Taken together, these synchrotron observations by FTIR and SXRF (Leskovjan et al., 2011), SXRF and XANES (Collingwood et al., 2005a; Antharam et al., 2012; Gallagher et al., 2012), and XANES and XMCD (Everett et al., 2014a,b), support the suggestion that mineralized iron deposits may prove a useful clinical marker of amyloid-mediated iron deposition (Meadowcroft et al., 2009; Petiet et al., 2011; Antharam et al., 2012), and they provide strong support for the hypothesis that interaction mechanisms between iron and aggregating Aβ, rather than necessarily measures of total iron concentration or co-localization of iron and amyloid, are critical to our understanding of Aβ-mediated toxicity in AD.

### APPROACHES TO STUDY DESIGN AND SAMPLE PREPARATION

As technical developments at synchrotron facilities have enabled and encouraged a growth in metallomics research over the past twenty years, some excellent progress has been made in understanding aspects of iron distribution and storage in the brain. However, this is a highly multidisciplinary area, requiring understanding of the biology, chemistry, and physics of the sample preparation, measurement techniques, and research question, in addition to appreciation of the neuroscience and (patho)physiology. Our aim in writing this review has been not only to showcase synchrotron insights into iron in neurodegenerative disorders, but also to provide a resource, through the cited literature, for investigators to draw upon when they are designing future synchrotron experiments.

Investigators often work with limited quantities of donated tissue specimens, with restricted information about the archive conditions or control over sample preparation. It is practical to work with small volumes of material because a high degree of sensitivity can be achieved with synchrotron experiments, but it is important to ensure that samples are properly selected, characterized, and matched in advance of the synchrotron experiment, and that all reasonable steps are taken to protect the integrity of the property being measured (e.g., avoiding steps that introduce contamination or alter the oxidation or mineral state of the iron being studied).

In the original XRF analysis of PD tissues by Earle, his concern that potassium may have leached from the tissue during storage (Earle, 1968) highlights a fundamental challenge in the analysis of trace metals in tissues. Sample storage and preparation may partially or completely alter the property to be analyzed, and careful experiment design is required to evaluate and minimize the scope for this. For example, fixation by prolonged immersion in a chemical solution such as formalin may be necessary to preserve components of the tissue, but it can have a detrimental effect on metal ions of interest where they are mobilized in fixative, and the extent of leaching varies depending on many factors including sample size, archival period, composition and pH of the fixing solution, and the bound states of the individual elements (ranging from tightly encapsulated within proteins or mineral deposits, to labile ions within the tissue). These factors will also determine the extent to which relevant chemical and mineral states are preserved. A consequence is that making comparisons between studies where some have used fixed tissue, and others have used fresh or frozen tissues, is not always viable. As metal ions are bound in a myriad number of ways, their propensity to migrate, to be washed out of samples, or altogether lost from tissues, can be expected to vary significantly depending on the experiment protocol, the microstructure of the tissue, and whether proportions of unbound or loosely bound metal ions are influenced by the disease condition being studied. The extent of iron leaching from archived chemically fixed tissue might be expected to depend on how strongly iron is bound in the tissue, raising the interesting question as to whether iron in healthy and diseased brains is equally prone to loss. We anticipate that the loosely bound iron implicated in neurodegenerative disorders (Kell, 2010) will be disproportionately lost in chemically fixed tissues.

Multi-modal imaging is especially valuable in addressing complex questions, and the sensitive site-specific analysis that can be performed in tissues with synchrotron techniques is of most value when observations can be directly related to broader histological findings in the same tissue. The non-destructive nature of complementary methods such as FTIR and SXRF are a great advantage, permitting multi-modal and repeated/multi-resolution analysis of sample regions, and one of the great advantages of synchrotron studies is the minimal sample preparation and handling required for most experiments. For example, cells can be grown directly onto the substrates to be imaged in air, and tissue may be cryo-sectioned and dried onto suitable support media with no further preparation other than to cover the section in a manner that complies with local health and safety guidelines.

Sample substrates are of great importance, as measurement sensitivity (such as in SXRF) precludes the use of standard microscope slides which contain iron in quantities significantly above SXRF detection limits. Slide coatings on any substrate can be problematic, as illustrated by Miller et al. (2006), unless they are of very high purity. Synthetic quartz slides are a practical option for many experiments if a rigid substrate is required. They do not permit X-ray transmission data to be obtained, but their chemical resistance facilitates subsequent histological staining. Thin polymeric films such as Ultralene^®^ have been used for many experiments due to their low scattering background, and they are especially useful where both transmission and reflection data are required. Silicon nitride membranes are a versatile alternative, particularly for work with cultured cells, and facilitate multi-modal imaging ranging from the hard X-ray region to the infra-red (Carter et al., 2010).

## CONCLUSION

In this review we have explored several ways in which synchrotron techniques have contributed to our understanding of iron and iron-binding metalloproteins in neurodegenerative disorders. This has included neuromelanin in PD, Aβ in AD, and the regional, cellular, and sub-cellular distribution of iron and other elements in health and disease. There is untapped scope to exploit synchrotron techniques in this field, especially multimodal analysis where complementary methods (such as FTIR and SXRF) can obtain correlations between proteins, metabolites, and elemental distributions at high spatial resolution. Analysis of concentration change alone cannot usually explain mechnisms or sufficiently inform treatment strategies, and insights into the inorganic chemistry aspects of the “iron question” in neurodegenerative disorders may yet be provided by synchrotron techniques that are still largely the preserve of chemists and physicists, such as XMCD (Everett et al., 2014a). The majority of studies to date have not included steps to preserve sample chemistry using cryogenic or anoxic conditions, but this may be necessary to address fundamental questions in neurodegenerative disorders. Additionally, many temporal aspects of iron trafficking within cells and tissues remain to be understood. The time-resolved experiments that can be achieved at synchrotrons may offer uniquely sensitive non-destructive opportunities to investigate real-time processes in living cells and tissues.

Synchrotron techniques offer tremendous potential to advance our understanding of iron dysmetabolism in neurodegenerative diseases, with unparalleled sensitivity, specificity, and temporal resolution making it practical to work with tiny analyte volumes, in highly dilute systems, performing unambigous speciation, and gaining a measure of many properties in a single or combined experiment at a given facility. The simplicity of the sample preparation techniques also offers excellent scope for complementary imaging. For example, SXRF has advanced our understanding of the relationship between iron and other elemental distributions in various regions of the brain in health and disease, and permitted identification of iron-rich structures in tissue so that they might subsequently be analyzed to determine oxidation states and biomineral form. Sub-cellular analysis has permitted direct investigation of the relationship between iron, other metal elements, dopamine, and neuromelanin, whereas lower-resolution spatial analysis has enabled direct correlation between iron maps and magnetic resonance imaging parameters to assist in the interpretation of clinical MRI measurement of brain iron.

Considering the work reported to date, the scale of which we have sought to capture in this review, it is evident that while certain questions have been studied in detail, some neurodegenerative disorders and/or brain regions have been largely overlooked, or only analyzed by a sub-set of the synchrotron techniques available. With this opportunity to learn more about the role of iron in neurodegenerative disorders, emphasis should be placed on experiment design, given the limited access to synchrotrons and to donations of human tissue for post-mortem analysis. This will ensure that with the burgeoning opportunities and growing interest in synchrotron research, carefully formed and precise questions are addressed to ensure effective advancement of our understanding of, and capacity to diagnose and treat, neurodegenerative disorders.

## LIST OF TERMS

The primary technical terms and acronyms used in this article are summarized below. A broader overview of techniques and terms is given in the review by McRae et al. (2009), and suggested guidelines for terminology in this field are provided elsewhere (Lobinski et al., 2010).

**Table:** 

FTIR	Fourier transform infrared spectroscopy
(LA)ICP-MS	(Laser-ablation) inductively coupled plasmamass spectrometry
PIXE	particle-induced X-ray emission
SAXS	small Angle X-ray Scattering
SIMS	secondary ion mass spectrometry
SXRF	synchrotron X-ray fluorescence
(E)XAFS	(Extended) X-ray absorption fine structure
XANES	X-ray absorption near edge spectroscopy
XAS	X-ray absorption spectroscopy
XCD	X-ray Circular Dichroism
XMCD	X-ray Magnetic Circular Dichroism
XSEM	X-ray secondary-emission microscopy

## AUTHOR CONTRIBUTIONS

Concept, first draft, and preparation of figures: Joanna F. Collingwood. Review and critical comment: Mark R. Davidson.

## Conflict of Interest Statement

The authors declare that the research was conducted in the absence of any commercial or financial relationships that could be construed as a potential conflict of interest.
